# Respiratory Effects of Fine and Ultrafine Particles from Indoor Sources—A Randomized Sham-Controlled Exposure Study of Healthy Volunteers

**DOI:** 10.3390/ijerph110706871

**Published:** 2014-07-04

**Authors:** Vanessa J. Soppa, Roel P. F. Schins, Frauke Hennig, Bryan Hellack, Ulrich Quass, Heinz Kaminski, Thomas A. J. Kuhlbusch, Barbara Hoffmann, Gudrun Weinmayr

**Affiliations:** 1 IUF-Leibniz Research Institute for Environmental Medicine, Auf’m Hennekamp 50, 40225 Düsseldorf, Germany; E-Mails: roel.schins@uni-duesseldorf.de (R.P.F.S.); frauke.hennig@IUF-Duesseldorf.de (F.H.); barbara.hoffmann@IUF-Duesseldorf.de (B.H.); gudrun.weinmayr@IUF-Duesseldorf.de (G.W.); 2 Air Quality and Sustainable Nanotechnology Unit, Institut für Energie- und Umwelttechnik (IUTA) e.V., Bliersheimer Straße 58–60, 47229 Duisburg, Germany; E-Mails: hellack@iuta.de (B.H.); quass@iuta.de (U.Q.); kaminski@iuta.de (H.K.); tky@iuta.de (T.A.J.K.); 3 Center for Nanointegration (CENIDE), University of Duisburg-Essen, Carl-Benz-Straße 199, 47057 Duisburg, Germany; 4 Medical Faculty, Heinrich-Heine-University of Düsseldorf, Universitätsstraße 1, 40225 Düsseldorf, Germany

**Keywords:** fine and ultrafine particles, controlled exposure, indoor sources, lung function

## Abstract

Particulate air pollution is linked to impaired respiratory health. We analyzed particle emissions from common indoor sources (candles burning (CB), toasting bread (TB), frying sausages (FS)) and lung function in 55 healthy volunteers (mean age 33.0 years) in a randomized cross-over controlled exposure study. Lung-deposited particle surface area concentration (PSC), size-specific particle number concentration (PNC) up to 10 µm, and particle mass concentration (PMC) of PM_1_, PM_2.5_ and PM_10_ were determined during exposure (2 h). FEV_1_, FVC and MEF_25__%–75%_ was measured before, 4 h and 24 h after exposure. Wilcoxon-rank sum tests (comparing exposure scenarios) and mixed linear regression using particle concentrations and adjusting for personal characteristics, travel time and transportation means before exposure sessions were performed. While no effect was seen comparing the exposure scenarios and in the unadjusted model, inverse associations were found for PMC from CB and FS in relation to FEV_1_ and MEF_25__%–75%_. with a change in 10 µg/m^3^ in PM_2.5_ from CB being associated with a change in FEV_1_ of −19 mL (95%-confidence interval:−43; 5) after 4 h. PMC from TB and PNC of UFP were not associated with lung function changes, but PSC from CB was. Elevated indoor fine particles from certain sources may be associated with small decreases in lung function in healthy adults.

## 1. Introduction

Exposure to ambient particulate matter (PM) is linked to increased morbidity and mortality with over a million premature deaths worldwide [1,2,3]. There is a clear link between PM and cardiovascular diseases as well as allergic and inflammatory conditions of the lung [1,4].

Increased levels of ambient PM air pollution have been associated with asthma exacerbations, increased respiratory symptoms, decreased lung function, and increased hospital admissions for respiratory symptoms due to asthma, chronic obstructive pulmonary disease, and other respiratory ailments as well as for cardiovascular disease [5,6,7].

Ultrafine particles (UFP) defined as particles with aerodynamic diameters below 100 nm received special attention recently [8] related to several characteristics which are thought to increase their pathogenic potential: High number concentrations, a high specific surface area and a high oxidative potential compared to larger particles at the same mass concentration [9,10,11]. They furthermore have a very high predicted deposition efficiency in the pulmonary region [12], can reach the alveolar space, penetrate the epithelium and might gain access to the pulmonary interstitium and systemic circulation [13,14].

To date the majority of studies investigated ambient particles, although in most industrialized countries people spend most of their time indoors [15] and significant emissions of fine and ultrafine particles leading to human exposure are caused by various indoor activities. These encompass for example candles burning, preparation of food, cleaning activities, use of electric engines or use of furnaces [16]. Afshari *et al.* examined 13 different indoor particle sources in a full-scale chamber [16]. They found a fast increase of UFP-concentrations shortly after the sources were activated with the highest UFP-concentrations when candles burning and noticeable concentrations when frying meat.

Several controlled exposure studies so far investigated the effects of diesel engine exhaust, wood smoke and strand board emissions on the respiratory and cardiovascular system [1,17,18,19,20,21,22,23], with inconsistent findings. Little is known about the effects of other emissions from specific indoor activities such as candles burning and preparing food.

In a controlled chamber exposure study, we investigate whether exposure to particles from candles burning, frying sausages and toasting bread as common indoor activities leads to changes in lung function in healthy volunteers. We perform an in-depth characterization of emitted particles combining established exposure metrics *i.e.*, size-specific particle mass concentration (PMC) with novel exposure metrics such as size-specific particle number concentration (PNC) and lung deposited surface area concentration (PSC) to detect a link between special characteristics of the emitted particles and lung function.

## 2. Experimental Section

The Effects of ultrafine Particles from Indoor Activities (EPIA) Study is a cross-over sham-controlled exposure study with healthy volunteers, integrating a detailed exposure characterization, the investigation of biological pathways with toxicological methods, and human health effect analyses. The study was performed with approval of the Ethics Committee of the Heinrich-Heine University of Düsseldorf, in accordance with the declaration of Helsinki, and with written informed consent of all participants.

Out of a convenience sample of 154 subjects who were interested in the study, a total number of 55 adult men and women met inclusion criteria and could be recruited for this study. Inclusion criteria comprised age between 18 and 79 years, speaking and understanding German, and being a non-smoker or ex-smoker for at least ten years. Predefined exclusion criteria were: existing or planned pregnancy (within the next six months), planned surgery in the following six months, occupational exposure to environmental tobacco smoke or air pollutants (e.g., welder, roadman, chemical worker, *etc.*), fatal disease in the end stage, heart attack or stroke in the past 3 months, uncontrolled arterial hypertension without medication, diabetes mellitus, chronic respiratory disease, chronic infectious disease and neurologic or mental disease (claustrophobia, psychosis).

All subjects were asked to abstain from alcohol and extreme physical exercise for 24 h and from caffeine drinks for at least 4 h before the beginning of each exposure. On the morning of the exposure days, participants arrived at the study center, having taken in only a light breakfast. Before medical examinations prior to the start of exposure, each participant was interviewed regarding his or her current health status and exposures prior to the study center visit. If a participant had a current infection or had taken anti-inflammatory drugs in the time interval between study center visits, the exposure was rescheduled. On average four participants were exposed on each exposure day.

Each exposure session lasted for two hours and took place in an air-conditioned laboratory room as exposure chamber. The chamber had an area and a volume of approximately 16 m^2^ and 48 m^3^, respectively. The walls were made from powder-coated sheet metal, windows from glass; the roof was covered with antistatic polyethylene film (10^9^–10^10^ ohms). Flow rate was approximately 250 m^3^/h. The air conditioning system worked in a circulating mode, hence not causing additional air exchange. Particle monitors operated during the exposure periods at a time resolution of minutes showed a relatively constant steady-state particle concentration, any fluctuation of concentrations was included in the exposure values. The sampling ports were installed close to the seats where the participants remained during exposure, distances ranged between 0.5 and 1.5 m. The vents (air conditioner and active air changer) ensured a good dispersion within the chamber. Tests made by handheld monitors (DISCmini, Matter Aerosol, Wohlen, Switzerland) at various locations around the participant’s seating area revealed concentrations comparable within the instrument uncertainties.

Participants were exposed on separate occasions at the same time of day at least two weeks apart. Participants were not blinded against the type of exposure because the particle sources were placed inside the exposure chamber so that the subjects could see and smell them. For the sham exposure with room air (RA), an air ventilator, was placed in the chamber. Participants did not exercise during exposure sessions so breathing patterns correspond to usual quiet indoor activities.

In the experimental exposure scenarios, participants were exposed to candle burning (CB), toasting bread (TB) and frying sausages (FS). Indoor sources were operated by staff being present continuously inside the chamber. Exposure scenarios were performed on two different exposure levels for each emission source. During CB, 20 white Christmas-tree candles (level 1, CB1) or 40 candles (level 2, CB2) were burning simultaneously. During the exposure scenario TB, bread was toasted with one (level 1, TB1) or two (level 2, TB2) identical time controlled 2-slice-toasters. The duration of each toasting period lasted up to 3 min. To ensure constant emission and in order to prevent the toaster from overheating, the toasting process was done alternately in different toasters of the same type. During the exposure scenario FS, three sausages per pan were fried simultaneously in one (level 1, FS1) or two (level 2, FS2) Teflon^®^-coated pans for 10 min without additional fat. To avoid extensive accumulation and burning of residues the pans were cooled down and cleaned after three cycles of frying. For this, the active emission was stopped transiently for about 10 min after each 0.5 h frying period.

Particle concentration in the exposure chamber was monitored continuously during each session to ensure consistent particle pollution levels for all participants and each exposure scenario and to calculate the individual cumulative particle exposure for each participant. Before a subject entered the exposure chamber the sources had already been activated to ensure that the particle concentration had reached the required equilibrium level. Size-specific number concentration of particles from 5.6 nm to 562 nm size range was monitored by a Fast Mobility Particle Sizer (FMPS, Model 3091, TSI Inc., Shoreview, MN, USA) and for particles from 0.5 to >10 µm with an Aerodynamic Particle Sizer (APS, Model 3321, TSI Inc.). Further, the alveolar deposited surface area particle concentration was measured in µm^2^/cm^3^, using a Nanoparticle Surface Area Monitor (NSAM, Model 3550, TSI Inc.). Additionally size-specific mass concentrations of PM_1_, PM_2.5_ and PM_10_ were calculated from particle size and number concentrations assuming spherical particles and a particle density of 1 g/cm^3^. During exposure, temperature and relative humidity was controlled and temperature maintained at 24 °C by a re-circulating air conditioning system installed in the chamber’s ceiling.

Forced vital capacity (FVC), forced expiratory volume at one second (FEV_1_) and averaged forced expiratory flow between expiration of 25% and 75% of total FVC (MEF_25__%–75%_) were assessed before, and 4 h as well as 24 h after exposure using the ndd Easy One-world-spirometer (NDD Medizintechnik AG, Zürich, Switzerland) with provided software (EasyWare Version 2.24.0.0, NDD Medizintechnik AG, Zürich, Switzerland). Spirometry was performed according to the American Thoracic Society/European Respiratory Society guidelines [24]. A minimum of three and a maximum of eight maneuvers were performed, and the values from the best acceptable maneuver, where the maximal sum of FVC and FEV_1_ was reached, were used [24].

Statistical Analyses were performed using SAS version 9.2 (SAS/STAT Software, SAS Institute, Inc., Cary, NC, USA) and R (R 2.15.2, Development Core Team, Vienna, Austria). Exposure and spirometry measurements are presented as a mean ± standard deviation. Changes in lung function post exposure (*t*_1_ = 4 h and *t*_2_ = 24 h) were quantified as the difference to pre-exposure (*t* = 0) values. To estimate exposure related changes in lung function (y_tik_) at each time point t computed for different indoor sources (*i* = 1 for candle burning, 2 for bread toasting and 3 for frying sausages) and exposure levels (*k* = 1, 2) in contrast to room air (RA) (*i* = 0), the difference-in-differences (DD, see equation) was calculated and tested using the paired Wilcoxon Rank-Sum test:

DD_t_ = (y_tik_ − y_0ik_) − (y_t0_ − y_00_), with *i* = 1–3 and *t* = 1, 2

The non-parametric Wilcoxon Rank-Sum test was used because the sample size was occasionally under 30 persons and lung function variables were not always normally distributed. Statistical testing was based on two-tailed tests with α = 0.05.

Additionally, in a multivariate analysis, we used the personal cumulative exposure to the particle metrics size-specific particle mass, particle number and surface area during the exposure sessions as independent variables and the intra-individual difference to *t*0 as dependent variable. For each exposure scenario *i.e.*, CB, TB and FS, respectively, separate linear mixed regression models with a random participant intercept were used to estimate the effect of PMC (for PM_10_, PM_2.5_, PM_1_), PNC (for UFP), and PSC. The effects for the two different time points were modeled with an interaction term incorporating an indicator variable for time point. In addition to the crude model, we adjusted for age, height, sex, temperature, humidity, travel time and means of transportation (full model). The models were fit via linear mixed models from the R language package.

## 3. Results

The study population shown in Table 1 consists of all participants who could be included in subsequent analyses. Altogether 55 participants were included, 28 men and 27 women. Due to air conditioning, the temperature inside the exposure chamber remained nearly constant at all exposure scenarios (Table 2). PNC was dominated by UFP (<100 nm), PSC by particles between 100–1000 nm in diameter and PMC by super-micron particles. The maximal number concentration for UFP was reached during candles burning (2,699,700 ± 205,900 particles/cm^3^) in level 2. Size-specific (<100 nm) PNC at level 2 was approximately twice as high as that for level 1 for all exposure scenarios. Contrary to that, PNC of PM_1_, PM_2.5_ and PM_10_ for CB and TB in level 1 was two to four times higher than in level 2, but not for FS. The mass-concentration for PM_1_, PM_2.5_, and PM_10_ at level 2 was on average at least one and a half time larger compared to level 1, except for PM_10_ at TB, where the PMC at level 2 was found to be smaller than at level 1. In general, the mass concentration was dominated by the contribution of ultrafine particles, which were a factor 10^4^ to 10^5^ higher in number than particles with size >500 nm. Hence, calculated PMC values in most cases follow the pattern of PNC for UFP. TB was an exception where a considerable decrease in large particle number concentration from level 1 (approx. 20 cm^−3^) to level 2 (approx. 4 cm^−3^) caused a decrease in PM10 PMC accompanied by a rising UFP number concentration. This is driven by one particular TB exposure session that was extreme in terms of the number of particles larger than 0.5 µm leading to high PM1, PM2.5 and PM10 exposure. Excluding the three participants of this exposure session reduces the average PM concentrations of TB level 1 down approximately half of the level 2 value.

The highest mass concentrations were observed during FS with the maximum at level 2. The surface-concentration was also roughly twice as high as at level 2 compared to level 1 for CB, TB and FS and was found to be nearly the same for all exposure scenarios at level 2.

**Table 1:** Personal and exposure session characteristics of 55 participants.

Characteristic	Measure
Age, years, mean (SD)	33.0 (16.6)
Born in Germany, *n* (%)	35 (64.8)
Male, *n* (%)	28 (50.9)
Weight, kg, mean (SD)	72.6 (14.0)
Height, cm, mean (SD)	174.3 (9.2)
Educational level, *n* (%)	
High School Graduation	42 (79.3)
Economic activity, *n* (%)	
Employed	25 (47.2)
Smoking status, *n* (%)	
Ex-smoker	3 (5.6)
Never-smoker	51 (94.4)
History of allergy	17 (32.7)
Residential exposures, *n* (%)	
Flat with local traffic	33 (61.1)
Flat located in street canyon	28 (51.9)
Flat with mildew	6 (11.1)
Damp flat	2 (3.7)
Temperature, C°, mean (SD)	23.8 (1.1)
Humidity, %, mean (SD)	34.8 (7.8)
Travel time, hours, mean (SD)	1.1 (0.5)
Means of transportation	
Car *n* (%)	106 (40.3)
Public transport *n* (%)	145 (55.1)
On foot, n (%)	2 (0.8)

Results of the lung function measurements before and after exposure are shown in Table 3. The number of evaluated manoeuvers differs between exposures because not all subjects participated in all exposure scenarios and not all lung function curves were acceptable. FEV_1_ decreased 4 h and 24 h after the exposure to RA and FS at level 1 and level 2, whereas it increased 4 h and 24 h after CB at level 1 and TB at level 2. A similar non-uniform pattern is apparent for FVC. Regarding FEV_1_/FVC, we observed mostly an increase after 4 h and 24 h after the particle exposure while the expiratory flow at 25%–75% of the vital capacity (MEF_25__%–75%_) shows a more heterogeneous picture with mainly increases but a stable value at RA (Table 3).

**Figure 1:**
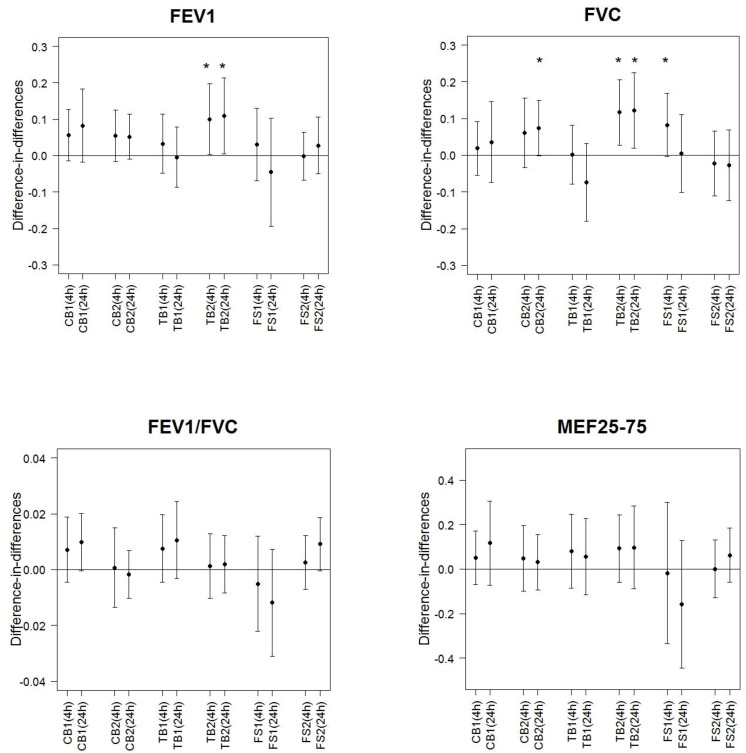
Difference-in-differences of lung function variables (FEV_1_ (L), FVC (L), FEV_1_/FVC (%), MEF_25__%–75%_ (L/s)) at 4 h and 24 h after exposure computed for different indoor sources and exposure levels contrasting to RA.

Notes: *: *p*-value < 0.05 from Wilcoxon signed rank test. RA: room air, CB: candles burning, TB: toasting bread, FS: frying sausages, 1: level 1, 2: level 2, (4 h) (24 h): 4 h and 24 h after exposure.

The means of the difference-in-differences for FEV_1_ were elevated or did not differ considerably from the null (Figure 1). For FVC the pattern was mostly similar. The mean difference-in-differences of FEV_1_/FVC showed no association and we did not observe any trend. The same applies with MEF_25__%–75%_ which showed, however, higher standard deviations. When taking into account multiple testing, none of the comparisons was statistically significant.

**Table 2:** Number of individuals exposed in each exposure scenario (N), mean and standard deviation (SD) of temperature, humidity and personal cumulative particle exposure.

Exposure Scenario	Temperature (C°)		Humidity (%)		PMC (µg/m^3^)					
					PM_1_		PM_2.5_		PM_10_	
	*N*	Mean ± SD	*N*	Mean ± SD	*N*	Mean ± SD	*N*	Mean ± SD	*N*	Mean ± SD
**Room air**	45	24.2 ± 0.4	45	30.5 ± 0.7	35	3.2 ± 0.5	35	4.7 ± 1.0	35	6.2 ± 2.0
**Candles burning**										
level 1	36	24.2 ± 0.4	36	37.1 ± 1.3	25	47.9 ± 9.2	25	52.6 ± 12.0	25	55.9 ± 13.7
level 2	38	24.7 ± 0.5	38	31.9 ± 1.3	38	79.3 ± 11.9	38	80.9 ± 13.8	38	83.7 ± 16.7
**Toasting bread**										
level 1	31	23.7 ± 0.5	31	36.2 ± 1.3	27	37.7 ± 7.0	27	62.6 ± 27.7	27	125.6 ± 87.1
level 2	32	23.6 ± 0.3	32	40.2 ± 2.5	35	79.9 ± 16.1	35	81.6 ± 16.6	35	84.6 ± 18.6
**Frying sausages**										
level 1	36	23.1 ± 0.4	36	38.5 ± 1.3	30	71.3 ± 28.2	36	84.4 ± 37.3	30	100.0 ± 51.9
level 2	36	23.0 ± 0.3	36	31.5 ± 1.3	36	207.8 ± 62.4	36	235.2 ± 81.4	36	296.9 ± 133.9
**Exposure Scenario**	**PSC**		**PNC (number/cm** **^3^)**							
	**LDSA (µm** **^2^/cm** **^3^)**		**<100 nm**		**0.5–1 µm**		**0.5–2.5 µm**		**PM_10_ (0.5–10 µm)**	
	***N***	**Mean** **±** **SD**	***N***	**Mean** **±** **SD**	***N***	**Mean** **±** **SD**	***N***	**Mean** **±** **SD**	***N***	**Mean** **±** **SD**
**Room air**	47	22.8 ± 2.1	47	0.3 ± 0.1 (* 10^4^)	35	2.3 ± 0.4	35	3.3 ± 0.8	35	3.4 ± 0.8
**Candles burning**										
level 1	36	2200.5 ± 137.8	36	190.8 ± 16.3 (* 10^4^)	25	6.2 ± 3.8	25	9.7 ± 5.7	25	9.9 ± 5.7
level 2	38	3839.6 ± 248.6	38	267.0 ± 20.6 (* 10^4^)	38	1.8 ± 2.3	38	2.7 ± 3.3	38	2.8 ± 3.5
**Toasting bread**										
level 1	35	1769.1 ± 318.0	34	90.4 ± 14.1 (* 104)	28	8.4 ± 3.8	28	19.0 ± 11.9	28	21.3 ± 13.9
level 2	35	3779.4 ± 577.0	35	155.8 ± 17.6 (* 104)	35	3.1 ± 0.5	35	4.3 ± 0.8	35	4.4 ± 0.8
**Frying sausages**										
level 1	36	1325.0 ± 432.6	36	31.1 ± 9.4 (* 104)	30	17.1 ± 10.2	30	24.3 ± 14.6	30	24.9 ± 15.2
level 2	36	3455.7 ± 660.0	36	60.7 ± 11.8 (* 104)	36	49.5 ± 30.1	36	65.6 ± 41.1	36	67.8 ± 42.8

Notes: PMC: particle mass concentration; PSC: particle surface concentration; PNC: particle number concentration; LDSA: lung deposited surface area.

**Table 3:** Number of measurements (N), mean and standard deviation (SD) of lung function variables before, and 4 h and 24 h after exposure to different indoor sources and exposure levels.

Exposure Scenario	FEV_1_ (L)						FVC (L)					
	Pre		Post 4 h		Post 24 h		Pre		Post 4 h		Post 24 h	
	*N*	Mean ± SD	*N*	Mean ± SD	*N*	Mean ± SD	*N*	Mean ± SD	*N*	Mean ± SD	*N*	Mean ± SD
RA	46	3.60 ± 0.84	45	3.51 ± 0.79	47	3.56 ± 0.78	46	4.47 ± 1.00	45	4.34 ± 0.94	47	4.43 ± 0.95
CB1	33	3.50 ± 0.79	35	3.55 ± 0.80	34	3.60 ± 0.83	33	4.35 ± 0.96	35	4.37 ± 0.94	34	4.45 ± 0.99
CB2	38	3.53 ± 0.89	38	3.55 ±0.91	36	3.56 ± 0.89	38	4.45 ± 1.09	38	4.42 ± 1.08	36	4.44 ± 1.08
TB1	33	3.50 ± 0.88	36	3.49 ± 0.82	34	3.48 ± 0.84	33	4.42 ± 1.00	36	4.34 ± 0.90	34	4.35 ± 0.93
TB2	35	3.54 ± 0.75	35	3.57 ± 0.79	35	3.60 ± 0.75	35	4.43 ± 0.91	35	4.43 ± 0.95	35	4.49 ± 0.92
FS1	35	3.50 ± 0.88	36	3.46 ± 0.85	35	3.43 ± 0.91	35	4.37 ± 1.00	36	4.35 ± 0.99	35	4.35 ± 1.04
FS2	34	3.57 ± 0.68	33	3.44 ± 0.70	33	3.50 ± 0.71	46	4.47 ± 0.83	33	4.26 ± 0.79	33	4.33 ± 0.86
**Exposure scenario**	**FEV_1_/FVC (%)**						**MEF_25__%__–75%_ (L/s)**					
	**Pre**		**Post 4 h**		**Post 24 h**		**Pre**		**Post 4 h**		**Post 24 h**	
	***N***	**Mean** **±** **SD**	***N***	**Mean** **±** **SD**	***N***	**Mean** **±** **SD**	***N***	**Mean** **±** **SD**	***N***	**Mean** **±** **SD**	***N***	**Mean** **±** **SD**
RA	46	0.81 ± 0.07	45	0.81 ± 0.07	47	0.81 ± 0.08	46	3.47 ± 1.22	45	3.47 ± 1.19	47	3.45 ± 1.17
CB1	33	0.81 ± 0.08	35	0.81 ± 0.08	34	0.81 ± 0.08	33	3.48 ± 1.25	35	3.60 ± 1.27	34	3.58 ± 1.27
CB2	38	0.79 ± 0.07	38	0.80 ± 0.07	36	0.80 ± 0.07	38	3.28 ± 1.18	38	3.41 ± 1.22	36	3.37 ± 1.22
TB1	33	0.79 ± 0.08	36	0.80 ± 0.08	34	0.80 ± 0.08	33	3.35 ± 1.37	36	3.48 ± 1.37	34	3.44 ± 1.41
TB2	35	0.80 ± 0.07	35	0.81 ± 0.07	35	0.80 ± 0.07	35	3.35 ± 1.16	35	3.48 ± 1.21	35	3.42 ± 1.10
FS1	35	0.80 ± 0.09	36	0.80 ± 0.09	35	0.79 ± 0.10	35	3.41 ± 1.34	36	3.41 ± 1.39	35	3.28 ± 1.39
FS2	34	0.80 ± 0.07	33	0.81 ± 0.08	33	0.81 ± 0.08	34	3.35 ± 1.06	33	3.33 ± 1.18	33	3.35 ± 1.12

Notes: FEV_1_: forced expiratory volume at 1 s, FVC: forced vital capacity, MEF_25__%–75%_: averaged forced expiratory flow between the full expiration of 25% and 75% of the total FVC, L: liter, L/s: liter per second; %: percent; RA: room air, CB1: candles burning level 1, CB2: candles burning level 2, TB1: toasting bread level 1, TB2: toasting bread level 2, FS1: frying sausages level 1, FS2: frying sausages level 2.

In mixed linear regression analyses, the crude model showed mostly positive associations for all three exposures for PMC and null associations for PSC and PNC (Table 4). In the fully adjusted model (full model), however, a 10 µg/m^3^ increase in PM_10_, PM_2.5_ and PM_1_ emitted from CB or FS was associated to decreases in FEV_1_. FVC tended to decrease after CB but not after FS or TB. The quotient FEV_1_/FVC decreased slightly after FS and after CB, but not after TB. Both CB and FS led to decreases in MEF_25__%–75%_, 4 h and 24 h after exposure, with confidence intervals excluding the null for FS (Table 4). Except for PSC from CB, no consistent effects were seen for PSC and PNC (Table 4). Because we aimed to refer to the same metric for all three exposure scenarios, e.g., 1 µg/m^3^, comparisons between particle metrics cannot be made directly but would need consideration of the emission range caused by the respective exposure scenarios. TB did not influence any lung function measures. Adjusting for “travel time” and “means of transportation” had by far the greatest impact on the effect estimates in comparison to the crude models (Table 4, details for changes of effect estimates when adding those variables in the last step to the final model can be found in Table S1 in the supplementary file). E.g., for MEF_25__%–75%_ and PM_10_, the effect estimates changed from −17 (95%-CI: −50; 16) to −23 (95%-CI: −70; 24) for CB and from 2 (95%-CI: −6; 10) to −11 (95%-CI: −21; −1) for FS when adding “travel time” and “means of transportation” to the model containing already the other covariates (Table S1, Supplementary File). The independent effect of the variables “means of transportation” and “travel time” was small and had large CIs. This is not surprising, as “means of transportation” only varied in 1–6 participants (on average 3) and average differences in “travel time” were small and had very large CIs, thereby limiting the power to detect a main effect. Even more than for the difference-in-differences, none of the comparisons was statistically significant when taking into account multiple testing.

**Table 4:** Mean effect estimates and 95% Confidence Intervall (CI) for changes (difference) associated with an increase in particulate metrics post 4 h and 24 h post exposure and for different exposure scenarios for PMC, PSC and PNC. Changes refer to an increase of 10 µg/m^3^ (PMC), 100 µm^2^/cm^3^ (PSC) and 10,000 number/cm^3^ (PNC).

Lung Function Measure	Exposure Scenario	PMC						PSC		PNC	
		PM_10_		PM_2.5_		PM_1_				<100 nm	
		Mean (95%-CI)		Mean (95%-CI)		Mean (95%-CI)		Mean (95%-CI)		Mean (95%-CI)	
		Post 4 h	Post 24 h	Post 4 h	Post 24 h	Post 4 h	Post 24 h	Post 4 h	Post 24 h	Post 4 h	Post 24 h
**FEV_1_ (m** **L** **)**	Candle burning										
	crude model	7 (−1; 15)	5 (−3; 13)	8 (0; 16)	5 (−3; 13)	8 (0; 16)	5 (−3; 13)	2 (0; 3)	1 (−1; 2)	0 (0; 0)	0 (0; 0)
	full model *	−18 (−40; 4)	−19 (−41; 3)	−19 (−43; 5)	−20 (−44; 4)	−22 (−47; 3)	−23 (−48; 2)	−13 (−20; −6)	−13 (−20; −6)	−1 (−2; 1)	−1 (−2; 1)
**FVC (m** **L** **)**	Candle burning										
	crude model	8 (−2; 18)	7 (−3; 17)	9 (−1; 19)	8 (−2; 18)	9 (−1; 19)	8 (−2; 18)	2 (0; 4)	0 (−2; 2)	0 (0; 0)	0 (0; 0)
	full model	−13 (−38; 12)	−12 (−39; 15)	−12 (−39; 15)	−11 (−38; 16)	−13 (−44; 18)	−12 (−43; 19)	−9 (−18; −1)	−10 (−18; −1)	−1 (−3; 0)	−1 (−3; 0)
	Toasting bread										
	crude model	0 (−2; 2)	−1 (−3; 1)	4 (−2; 10)	1 (−5; 7)	14 (4; 24)	14 (4; 24)	3 (1; 5)	2 (0; 5)	1 (0; 1)	0 (0; 1)
	full model	0 (−2; 2)	−2 (−10; 6)	1 (−7; 9)	−2 (−16; 12)	0 (−22; 22)	0 (−21; 19)	1 (−7; 9)	1 (−7; 8)	1 (−2; 4)	1 (−2; 4)
	Frying sausages										
	crude model	0 (−2; 2)	0 (−2; 2)	0 (−4; 4)	0 (−4; 4)	1 (−3; 5)	0 (−4; 4)	0 (−2; 3)	−1 (−3; 1)	0 (−1; 2)	−1 (−2; 1)
	full model	2 (−2; 6)	1 (−3; 5)	3 (−3; 9)	2 (−4; 8)	5 (−1; 11)	4 (−4; 12)	3 (−1; 7)	2 (−3; 7)	4 (−1; 8)	3 (−2; 8)
	
**FEV_1_/FVC (%); Percentage Point** * **1000**	Candle burning										
	crude model	0 (−2; 2)	0 (−2; 2)	0 (−2; 2)	0 (−2; 2)	0 (−2; 2)	0 (−2; 2)	0 (0; 0)	0 (0; 0)	0 (0; 0)	0 (0; 0)
	full model	−2 (−6; 2)	−3 (−7; 1)	−3 (−7; 1)	−3 (−7; 1)	−3 (−7; 1)	−4 (−8; 0)	−1 (−2; 0)	1 (−2; 0)	0 (0; 0)	0 (0; 0)
	Toasting bread										
	crude model	0 (0; 0)	0 (0; 0)	0 (0; 0)	0 (0; 0)	0 (−2; 2)	0 (−2; 2)	0 (0; 0)	0 (0; 0)	0 (0; 0)	0 (0; 0)
	full model	0 (0; 0)	0 (0; 0)	0 (0; 0)	0 (−2; 2)	−1 (−3; 1)	−1 (−3; 1)	−1 (−2; 0)	−1 (−2; 0)	0 (0; 0)	0 (0; 0)
	Frying sausages										
	crude model	0 (0; 0)	0 (0; 0)	0 (0; 0)	0 (0; 0)	0 (0; 0)	0 (0; 0)	0 (0; 0)	0 (0; 1)	0 (0; 0)	0 (0; 0)
	full model	−1 (−1; −1)	−1 (−1; −1)	−2 (−4; 0)	−2 (−4; 0)	−2 (−4; 0)	−2 (−4; 0)	−1 (−2; 0)	0 (−1; 0)	−1 (−2; 0)	−1 (−1; 0)
**MEF_25_** **_%_** **_–75%_** ** (m** **L** **/s)**	Candle burning										
	crude model	9 (−7; 25)	−3 (−19; 13)	10 (−6; 26)	−2 (−18; 14)	10 (−6; 26)	−2 (−20; 16)	2 (−1; 6)	1 (−3; 4)	0 (0; 1)	0 (0; 1)
	full model	−23 (−70; 24)	−37 (−84; 10)	−26 (−75; 23)	−39 (−88; 10)	−31 (−84; 22)	−45 (−100; 10)	−13 (−28; 2)	−14 (−29; 1)	1 (−2; 4)	1 (−2; 4)
	Toasting bread										
	crude model	1 (−3; 5)	0 (−4; 4)	5 (−5; 15)	0 (−10; 10)	11 (−7; 29)	2 (−16; 20)	3 (−1; 7)	1 (−3; 5)	1 (0; 2)	0 (0; 1)
	full model	1 (−3; 5)	0 (−14; 14)	1 (−11; 13)	−6 (−30; 18)	−14 (−49; 21)	−22 (−57; 13)	−8 (−22; 6)	−9 (−23; 5)	1 (−4; 6)	1 (−4; 6)
	Frying sausages										
	crude model	0 (−6; 6)	2 (−4; 8)	−1 (−9; 7)	1 (−7; 9)	0 (−8; 8)	2 (−8; 12)	0 (−5; 6)	1 (−4; 7)	1 (−3; 4)	1 (−3; 4)
	full model	−11 (−21; −1)	−8 (−20; 4)	−23 (−37; −9)	−20 (−36; −4)	−22 (−38; −6)	−19 (−39; 1)	−8 (−18; 3)	−5 (−17; 7)	−7 (−18; 5)	−5 (−17; 6)

Notes: * adjusted for age, height, sex, temperature, humidity, travel time and means of transportation; Abbreviations: FEV_1_: forced expiratory volume at 1 s, FVC: forced vital capacity, MEF_25__%–75%_: averaged forced expiratory flow between the full expiration of 25% and 75% of the total FVC, CI: confidence interval; marked in bold: effect estimates where CI did not include the null effect. PMC: particle mass concentration; PSC: particle surface concentration; PNC: particle number concentration; LDSA: lung deposited surface area.

## 4. Discussion

Based on several inverse associations from the linear regression analysis adjusted for transport means and other covariates, our study indicates a possible association of short-term exposure to fine and ultrafine particles emitted from common indoor sources with small decreases in lung function in healthy adults. Moreover, size-specific particle mass concentrations may be more consistently associated with decreases in lung function than novel metrics such as surface area or size-specific particle number. Measured changes in indoor sources differed between type of exposure source with candle burning and frying sausages showing small effect measurement sizes whereas toasting bread did not show any association on lung function. However results have to be viewed also with caution because of the explorative nature of the analysis of several PM-metrics implying multiple comparisons and it can therefore not be excluded that they are in line with null-findings. Nevertheless, results from the adjusted model should be taken as point of reference because the important changes in effect estimates point to presence of confounding in the crude models. Overall, elevated indoor fine particles from certain sources may therefore be associated with small decreases in lung function in healthy adults.

The observed associations of particle metrics on lung function were visible more often for size-specific particle mass in contrast to novel metrics such as particle surface area or size-specific particle number concentration. Associations were mostly observed for PM_1_ and PM_2.5_, while PSC from CB, only, was associated with lung function outcomes. The association of UFP (PNC (<100 nm)) showed a trend towards a decrease with MEF_25__%__–75%_, only. This seems to be in contrast to prior toxicological findings, showing stronger associations for UFP than for fine particles of the same chemical composition and on an equal mass basis [11]. However, because we aimed to have the same metric for all three exposure scenarios, such as e.g., 1 µg/m^3^, comparisons between particle metrics cannot be made directly and have to be viewed with caution.

The results of the full regression model, adjusted for personal characteristics as well as for time in travel and means of transportation on the morning before exposure, showed some consistent short-term decreases in lung function for candle burning and frying sausages. In contrast, the crude models did not exhibit these effects. Similarly, the analysis of difference-in-differences, directly comparing changes to pre-exposure values for each exposure scenario with changes after room air exposure, had no consistent effect on any lung function variable. This is may be due to better resolution of exposure with individual-specific PM-concentrations and points to the potential importance of taking the individual (pre-)exposure to traffic-related air pollution prior to the controlled exposure into account, especially given the large catchment area of our participants (comprising an area of approximately 1000 km^2^), and the intra-individual changes in means of transportation (*i.e.*, change from public transportation to private cars), travel time and therefore ambient air pollution exposure before the start of the controlled exposure. Nevertheless, one should be cautious given that the confidence intervals generally increased considerably after adjustment, concomitant with an important change of the effect estimates.

Our results show most consistent associations for MEF_25__%__–75%_, which is an indicator for small airways obstruction, compared to FEV_1_ and FVC, showing that small airways may be more affected by the investigated particle emissions than larger airways. Other studies indicate a mild airway inflammation after short-term exposure to air pollution [18,19,20,21,22,23]. Several controlled chamber studies with wood smoke and emissions from oriented strand board showed mild systemic and pulmonary inflammation but no effects on those lung function indices, which seem to be less sensitive to changes in small airways such as PEF (peak expiratory flow), FEV_1_ and FVC [21,22,23]. Controlled exposure studies investigating the effect of diesel exhaust (DE) particles also showed airway inflammation, however only effects on larger airways were investigated—showing no effect—and results for small airways were not reported [17,18,19]. After short-term (3 h) DE exposure in comparison to filtered air, no changes of FEV_1_ and FVC were observed [20], similar to Nightingale *et al.*, 2000 [18], and Stenfors *et al.* [19]. In the latter two studies controlled two hour exposure to DE particles (200 µg/m^3^ PM_10_ [18] and 108 µg/m^3^ PM_10_ [19]) did not affect FEV_1_ and FVC whereas it induced airway inflammation [18,19]. In contrast, Xu *et al.* showed that 75 min exposure to diesel particles (PM_1_: 300 µg/m^3^) leads to a temporary decline in PEF in healthy subjects, along with self-reported irritations in upper airways [20]. Additionally, the increase in leukocyte cell counts in peripheral blood indicates that these levels of DE exposure also cause a systemic inflammatory response [20]. Despite the lack of consistent changes in large airway-specific lung function indices after short-term controlled exposure to wood smoke and DE particles, airway inflammation could be observed in all of these studies, indicating that emitted particles are harmful to the respiratory system. Lung function measurements, especially the measurement of FEV_1_ and FVC, may be less sensitive for picking up small effects of short-term exposures in healthy subjects and more sensitive indices or instruments might be necessary.

Biological pathways for the observed effects may lead to constriction due to either include airway inflammation or neurogenic responses. Depending on the size of inhaled particles, they deposit in the nasopharynx, enter the main bronchi or even reach the alveoli. PM inhalation can cause inflammation and oxidative stress response within the lungs, detectable through an increase of e.g., cytokines and white blood cells. The inflammatory response will lead to a swelling of the bronchial mucosa, decreasing the overall diameter of the small airways. Furthermore, it can lead to an activation of bronchial smooth muscle cells, causing a subclinical constriction of the small airways [1]. Chemical stimuli have been shown to stimulate bronchopulmonary C-fiber afferents leading also to constriction of airways and increased mucus secretion [25].

Several panel or controlled exposure studies investigated asthmatic participants, as these are believed to be especially susceptible to the effects of inhaled fine and ultrafine particles. Strong evidence for short-term effects on lung function has come from panel studies in asthmatic children [26,27,28,29,30]. Boman *et al.*, reviewed panel studies on pulmonary effects of people living in areas where the exposure was dominated by residential wood combusting, summarizing that the most information was only found for acute asthma in relation to particulate matter with an aerodynamic diameter of <10 μm [27]. The results of the studies of Vedal *et al.* [30] and Koenig *et al.* [26] showed that asthmatic children, living in an area with high particulate concentrations coming predominantly from residential wood burning, had a lowered lung function, e.g., decreased PEF (0.55 L/min) per increase of 10 µg/m^3^ PM_10_ [30] and decreased FEV_1_ (34 mL) and FVC (37 mL) per increase of 20 µg/m^3^ PM_2.5_ [26], in contrast to non-asthmatic children. In a British study, participants with mild asthma were exposed to a two hour exposure on a busy street with a high traffic load of heavy-duty Diesel engines in London (Oxford Street) and a two hour exposure in a park [31]. The authors observed reduced lung function measured as MEF_25__%__–75%_, as well as decreases in FEV_1_, and FVC in relation to ultrafine particles, and less consistently for PM_2.5_, which clearly reduced only MEF_25__%–75%_. In contrast to these studies on presumably more susceptible asthmatic patients, we studied changes in healthy participants. Even though the participants in our study were thought to be less susceptible than asthmatic patients, we found effect estimates, that although they are relatively instable, compare well in size with those found in asthmatics [26,27,30,31].

In our exposure assessment the concentration levels (PMC, PSC and PNC) at CB, TB and FS differed strongly to the sham exposure. For size-specific PMC, exposures were 10 to 50 times higher and for size-specific PNC they were 100 to 900 times higher than during room air exposure. For all exposure metrics and for all exposure session we could achieve a sufficient contrast between level 1 and level 2, allowing exposure-dependent analyses. In each of our exposure scenarios we observed very high number concentrations of UFPs. Similar to Afshari *et al.*, the highest particle number concentrations were reached during candles burning at level 2 and were dominated by UFPs [16]. Interestingly, regarding the PNC during FS, there was a higher amount of particles >10 µm (data not shown) compared to BC and TB and PMC during FS was dominated by particles of PM_2.5_ and PM_10_, possibly due to fatty droplets which form larger particles.

Despite the consistently high concentration levels during all exposure scenarios, observed associations with lung function seemed to differ between exposures, which may reflect different source-specific constituents and resulting differential toxicity of the emitted particles. Toxicological investigations have demonstrated that, depending on their chemical composition and source, the toxic potency of outdoor PM samples of similar size fractions can vary considerably [32,33,34].

In our study we specifically investigated effects of indoor sources of fine and ultrafine particles. Even though a large part of particles found indoors originate from outdoors (traffic, industry, agriculture, earth crustal material), indoor sources can potentially result in relevant personal cumulative exposures in rooms with insufficient ventilation. Particle mass concentrations for PM_10_ and PM_2.5_ resulting from candles burning and frying sausages were 2 to 12 times higher during chamber exposure than ambient daily mean concentrations, measured at an urban background location in the highly industrialized and urbanized Ruhr area in Germany [35] and comparable with indoor measurements during daily activities. For example, Wallace *et al.*, 2003, found out that mean indoor PM_2.5_ over a 14 days period was 31 μg/m^3^ in homes with cooking and 23.5 μg/m^3^ in homes without cooking, with an average increase of about 3.5 μg/m^3^ due to the average number of frying events (nine per week) [36]. Cumulative exposure during a time period of several days might therefore well lead to similar cumulative exposures as in our study. The fact that the reported lung function changes are relatively mild given the very high PNC highlights that extrapolations from outdoor studies to indoor studies (and *vice versa*) should generally not be undertaken.

Our study has several limitations. Associations could be observed only in the multivariate regression analysis but not in the unadjusted results of the analysis of difference-of-differences between exposure scenarios. While the adjustment for relevant covariates helps to address potential confounding, confidence intervals increased considerably. Furthermore, the advantage of investigating several different exposure metrics is counterbalanced by the possibility to have chance findings due to multiple comparisons. Indeed, given the high number of comparisons, our results could, in the worst case, be the result of chance findings. This is a clear drawback of our analyses which reflects therefore the results of an exploratory analysis and should by no means be interpreted in terms of conclusive statistical tests. Due to sick leave, premature study discontinuation or other personal reasons not all participants received all exposure scenarios. To create controlled and standardized exposure scenarios which are as uniform as possible across study participants, we were not able to eliminate differences to real-life exposures in a common household. The candles for example have been replaced before burning down, decreasing soot emissions and the sausages were fried in a pan without fat. One major limitation is that during the exposure of the participants we did not measure gases that were emitted at the same time and are often irritants and therefore could have affected lung function. Spurious results could result if these gases correlated strongly with emitted particle concentrations. However, measurements during the exposure characterization with a proton-transfer-reaction mass spectrometer (PTR-MS, Ionicon) showed sub-ppm levels for all detectable gas-phase compounds (e.g., aldehydes, alcohols, organic acids, NO_x_). We have not measured O_3_ that could also potentially alter lung function. It cannot be excluded that the exposures under real-life conditions could provoke different effects, all the more as exposure levels may be considerably lower than those that were chosen for the present study. Regardless of the type of exposure, the exposure time of two hours was short and possibly prevented us from observing more clear and consistent effects on lung function variables. The selected time points for the examination of lung function have been selected on the basis of previous studies who also measured three to 6 h and 22 to 24 h after exposure, respectively [18,19,23,31]. We cannot rule out that different time points of measurement, such as directly after exposure, would have led to more pronounced effects on lung function.

The strength of our study is a detailed exposure characterization, including continuous measurements of number size distribution, size-specific mass concentrations and surface concentrations, enabling us to assess personal cumulative exposures for established and novel particle metrics. This improves exposure assessment over the mere classification as CB, TB and FS that was used for the test. We achieved a high exposure contrast between the sham exposure and the experimental exposures for all particle metrics, which increased our power to detect even small, clinically not relevant effects.

## 5. Conclusions

In our study of short-term exposure of healthy adults, we found some associations of fine particles emitted from common indoor sources in the fully adjusted model that also took account of the means of transportation on the day of the exam. However, no consistent effect was seen comparing merely the exposure scenarios or in the unadjusted or partially adjusted model, In the fully adjusted model, associations of fine particles emitted from candle burning and frying sausages with small negative changes in lung function variables were observed. Toasting bread showed no association with lung function. The novel particle metric size-specific PNC was not associated with reduced lung function and PSC showed associations only for candles burning. Common household activities such as candles burning, toasting bread and frying sausages may cause particle mass concentrations indoors, which can possibly cause transient negative changes in respiratory function. Thus, elevated indoor fine particles from certain sources may be associated with small decreases in lung function in healthy adults. However, because we cannot exclude the possibility of our results being in line with chance findings from multiple comparisons, further in depth investigations are necessary to corroborate these results and to investigate potential biological pathways involving local pulmonary inflammation and more sensitive markers of small airway constriction.

## Supplementary Files

Supplementary File 1 Supplementary Information (PDF, 150 KB)
