# Correction: Soppa, V.J., *et al.* Respiratory Effects of Fine and Ultrafine Particles from Indoor Sources—A Randomized Sham-Controlled Exposure Study of Healthy Volunteers *Int. J. Environ. Res. Public Health* 2014, *11*, 6871–6889

**DOI:** 10.3390/ijerph111010362

**Published:** 2014-10-07

**Authors:** Vanessa J. Soppa, Roel P. F. Schins, Frauke Hennig, Bryan Hellack, Ulrich Quass, Heinz Kaminski, Thomas A. J. Kuhlbusch, Barbara Hoffmann, Gudrun Weinmayr

**Affiliations:** 1 IUF - Leibniz Research Institute for Environmental Medicine, Auf`m Hennekamp 50, 40225 Düsseldorf, Germany; E-Mails: Roel.Schins@uni-duesseldorf.de (R.P.F.S.); Frauke.Hennig@IUF-Duesseldorf.de (F.H.); Barbara.Hoffmann@IUF-Duesseldorf.de (B.H.); Gudrun.Weinmayr@uni-duesseldorf.de (G.W.); 2 Air Quality & Sustainable Nanotechnology Unit, IUTA e.V., Bliersheimer Straße 58–60, 47229 Duisburg, Germany; E-Mails: hellack@iuta.de (B.H.); quass@iuta.de(U.Q.); kaminski@iuta.de (H.K.); tky@iuta.de (T.A.J.K.); 3 Center for Nanointegration (CENIDE), University of Duisburg-Essen, Carl-Benz-Straße 199, 47057 Duisburg, Germany; 4 Medical Faculty, Heinrich-Heine-University of Düsseldorf, Universitätsstraße 1, 40225 Düsseldorf, Germany

The authors wish to make the following amendments to their paper published in *International Journal of Environmental Research and Public Health* [1]:

1. Page 6880, Table 3: “FEV_1_/FVC[%]” should read “FEV_1_/FVC”.
2. Page 6880, Table 4: “10,000 Number/cm^3^ (PNC)” should read “10,000 /cm^3^ (PNC)”, “FEV1/FVC (%); Percentage Point * 1000” should read “FEV1/FVC × 1000”. The items for FEV1 for Toasting Bread and Frying Sausages were missing. The correct Table 4 should be:

**Table 4:** Mean effect estimates and 95% Confidence Interval (CI) for changes (difference) associated with an increase in particulate metrics post 4 h and 24 h post exposure and for different exposure scenarios for PMC, PSC and PNC. Changes refer to an increase of 10 µg/m^3^ (PMC), 100 µm^2^/cm^3^ (PSC) and 10,000 /cm^3^ (PNC).

Lung function measure	Exposure scenario	PMC						PSC				PNC		
		PM_10_		PM_2.5_		PM_1_						<100 nm		
		Mean (95%-CI)		Mean (95%-CI)		Mean (95%-CI)		Mean (95%-CI)				Mean (95%-CI)		
		Post 4 h	Post 24 h	Post 4 h	Post 24 h	Post 4 h	Post 24 h	Post 4 h		Post 24 h		Post 4 h	Post 24 h	
**FEV_1_ (mL)**	Candle burning													
	crude model *	7 (−1; 15)	5 (−3; 13)	8 (0; 16)	5 (−3; 13)	8 (0; 16)	5 (−3; 13)	2 (0; 3)		1 (−1; 2)		0 (0; 0)	0 (0; 0)	
	full model *	−18 (−40; 4)	−19 (−41; 3)	−19 (−43; 5)	−20 (−44; 4)	−22 (−47; 3)	−23 (−48; 2)	−13 (−20; −6)		−13 (−20; −6)		−1 (−2; 1)	−1 (−2; 1)	
	Toasting bread													
	crude model	0 (−2; 2)	0 (−2; 2)	4 (−2; 10)	2 (−4; 8)	11 (1; 21)	10 (0; 20)	3 (1; 5)		2 (0; 4)		1 (0; 1)	0 (0; 1)	
	full model	0 (−2; 2)	0 (−8; 8)	1 (−5; 7)	−2 (−16; 12)	−6 (−26; 14)	−7 (−27; 13)	−2 (−10; 5)		−3 (−10; 5)		1 (-2; 4)	1 (−2; 4)	
	Frying sausages													
	crude model	0 (−2; 2)	1 (−1; 3)	0 (−4; 4)	1 (−3; 5)	1 (−3; 5)	2 (−2; 6)	1 (−2; 3)		0 (−2; 3)		0 (−1; 2)	0 (−1; 2)	
	full model	−3 (−7; 1)	−2 (−8; 4)	−6 (−12; 0)	−5 (−13; 3)	−5 (−13; 3)	−3 (−11; 5)	−1 (−6; 4)		0 (−5; 5)		0 (−5; 5)	0 (−5; 6)	
**FVC (mL)**	Candle burning													
	crude model	8 (−2; 18)	7 (−3; 17)	9 (−1; 19)	8 (−2; 18)	9 (−1; 19)	8 (−2; 18)	2 (0; 4)		0 (−2; 2)		0 (0; 0)	0 (0; 0)	
	full model	−13 (−38; 12)	−12 (−39; 15)	−12 (−39; 15)	−11 (−38; 16)	−13 (−44; 18)	−12 (−43; 19)	−9 (−18; −1)		−10 (−18; −1)		−1 (−3; 0)	−1 (−3; 0)	
	Toasting bread													
	crude model	0 (−2; 2)	−1 (−3; 1)	4 (−2; 10)	1 (−5; 7)	14 (4; 24)	14 (4; 24)	3 (1; 5)		2 (0; 5)		1 (0; 1)	0 (0; 1)	
	full model	0 (−2; 2)	−2 (−10; 6)	1 (−7; 9)	−2 (−16; 12)	0 (−22; 22)	0 (−21; 19)	1 (−7; 9)		1 (−7; 8)		1 (−2; 4)	1 (−2; 4)	
	Frying sausages													
	crude model	0 (−2; 2)	0 (−2; 2)	0 (−4; 4)	0 (−4; 4)	1 (−3; 5)	0 (−4; 4)	0 (−2; 3)		−1 (−3; 1)		0 (−1; 2)	−1 (−2; 1)	
	full model	2 (−2; 6)	1 (−3; 5)	3 (−3; 9)	2 (−4; 8)	5 (−1; 11)	4 (−4; 12)	3 (−1; 7)		2 (−3; 7)		4 (−1; 8)	3 (−2; 8)	
**FEV_1_/FVC × 1000**	Candle burning													
	crude model	0 (−2; 2)	0 (−2; 2)	0 (−2; 2)	0 (−2; 2)	0 (−2; 2)	0 (−2; 2)	0 (0; 0)	0 (0; 0)		0 (0; 0)			0 (0; 0)
	full model	−2 (−6; 2)	−3 (−7; 1)	−3 (−7; 1)	−3 (−7; 1)	−3 (−7; 1)	−4 (−8; 0)	−1 (−2; 0)	1 (−2; 0)		0 (0; 0)			0 (0; 0)
	Toasting bread													
	crude model	0 (0; 0)	0 (0; 0)	0 (0; 0)	0 (0; 0)	0 (−2; 2)	0 (−2; 2)	0 (0; 0)	0 (0; 0)		0 (0; 0)			0 (0; 0)
	full model	0 (0; 0)	0 (0; 0)	0 (0; 0)	0 (−2; 2)	−1 (−3; 1)	−1 (−3; 1)	−1 (−2; 0)	−1 (−2; 0)		0 (0; 0)			0 (0; 0)
	Frying sausages													
	crude model	0 (0; 0)	0 (0; 0)	0 (0; 0)	0 (0; 0)	0 (0; 0)	0 (0; 0)	0 (0; 0)	0 (0; 1)		0 (0; 0)			0 (0; 0)
	full model	−1 (−1; −1)	−1 (−1; −1)	−2 (−4; 0)	−2 (−4; 0)	−2 (−4; 0)	−2 (−4; 0)	−1 (−2; 0)	0 (−1; 0)		−1 (−2; 0)			−1 (−1; 0)

Notes: * adjusted for age, height, sex, temperature, humidity, travel time and means of transportation. Abbreviations: FEV_1_: forced expiratory volume at 1 s, FVC: forced vital capacity, MEF_25%–75%_: averaged forced expiratory flow between the full expiration of 25% and 75% of the total FVC, CI: confidence interval; marked in bold: effect estimates where CI did not include the null effect. PMC: particle mass concentration; PSC: particle surface concentration; PNC: particle number concentration; LDSA: lung deposited surface area.

The authors would like to apologize for any inconvenience caused to readers by these changes.
