# Supplementary material for: Respiratory Effects of Fine and Ultrafine Particles from Indoor Sources—A Randomized Sham-Controlled Exposure Study of Healthy Volunteers
Source: Int J Environ Res Public Health. 2014 Jul 4;11(7):6871–89. doi: 10.3390/ijerph110706871 (PMC4113851; doi:10.3390/ijerph110706871)
Supplement: Supplementary File 1 — Supplementary Information (PDF, 150 KB) [file ijerph-11-06871-s001.pdf]

# Respiratory Effects of Fine and Ultrafine Particles from Indoor Sources—A Randomized Sham-Controlled Exposure Study of Healthy Volunteers

**Table S1.** Mean effect estimates and 95% Confidence Intervall (CI) for changes (difference) associated with an increase in particulate metrics post 4 h and 24 h post exposure and for different exposure scenarios for PMC, PSC and PNC. Changes refer to an increase of 10 μg/m$^3$ (PMC), 100 μm$^2$/cm$^3$ (PSC) and 10,000.

| Lung Function Measure | Exposure Scenario | PMC | | | | | | PSC | | PNC | |
|---|---|---|---|---|---|---|---|---|---|---|---|
| | | $PM_{10}$ | | $PM_{2.5}$ | | $PM_1$ | | | | <100 nm | |
| | | Mean (95%-CI) | | Mean (95%-CI) | | Mean (95%-CI) | | Mean (95%-CI) | | Mean (95%-CI) | |
| | | Post 4 h | Post 24 h | Post 4 h | Post 24 h | Post 4 h | Post 24 h | Post 4 h | Post 24 h | Post 4 h | Post 24 h |
| FEV$_1$ (mL) | **Candle burning** | | | | | | | | | | |
| | crude model | 7 (−1; 15) | 5 (−3; 13) | 8 (0; 16) | 5 (−3; 13) | 8 (0; 16) | 5 (−3; 13) | 2 (0; 3) | 1 (−1; 2) | 0 (0; 0) | 0 (0; 0) |
| | model 1 | −3 (−19; 13) | −5 (−21; 11) | −2 (−18; 14) | −4 (−20; 12) | (−16; 16) | −3 (−19; 13) | −1 (−4; 2) | −2 (−5; 1) | 0 (−1; 1) | 0 (−1; 0) |
| | model 2 | −8 (−24; 8) | −10 (−26; 6) | −7 (−23; 9) | −10 (−28; 8) | −6 (−22; 10) | −8 (−24; 8) | −1 (−4; 2) | −2 (−5; 1) | 0 (−1; 1) | 0 (−1; 0) |
| | full model * | −18 (−40; 4) | −19 (−41; 3) | −19 (−43; 5) | −20 (−44; 4) | −22 (−47; 3) | −23 (−48; 2) | **−13 (−20; −6)** | **−13 (−20; −6)** | −1 (−2; 1) | −1 (−2; 1) |
| | **Toasting bread** | | | | | | | | | | |
| | crude model | 0 (−2; 2) | 0 (−2; 2) | 4 (−2; 10) | 2 (−4; 8) | **11 (1; 21)** | 10 (0; 20) | **3 (1; 5)** | 2 (0; 4) | 1 (0; 1) | 0 (0; 1) |
| | model 1 | 0 (−2; 2) | −1 (−3; 1) | 0 (−6; 6) | −2 (−8; 4) | 6 (−8; 20) | 5 (−9; 19) | **4 (1; 7)** | 3 (0; 6) | 1 (0; 2) | 1 (0; 2) |
| | model 2 | 0 (−2; 2) | 0 (−2; 2) | 1 (−5; 7) | −1 (−7; 5) | 5 (−9; 19) | 4 (−10; 18) | 3 (0; 7) | 3 (−1; 6) | 1 (0; 2) | 1 (0; 2) |
| | full model | 0 (−2; 2) | 0 (−8; 8) | 1 (−5; 7) | −2 (−16; 12) | −6 (−26; 14) | −7 (−27; 13) | −2 (−10; 5) | −3 (−10; 5) | 1 (−2; 4) | 1 (−2; 4) |
| | **Frying sausages** | | | | | | | | | | |
| | crude model | 0 (−2; 2) | 1 (−1; 3) | 0 (−4; 4) | 1 (−3; 5) | 1 (−3; 5) | 2 (−2; 6) | 1 (−2; 3) | 0 (−2; 3) | 0 (−1; 2) | 0 (−1; 2) |
| | model 1 | 1 (−3; 5) | 1 (−3; 5) | 1 (−3; 5) | 2 (−2; 6) | 1 (−3; 5) | 3(−1; 7) | 1 (−2; 4) | 1 (−2; 4) | 2 (−1; 4) | 1 (−1; 4) |
| | model 2 | 1 (−3; 5) | 2 (−2; 6) | 1 (−3; 5) | 2 (−2; 6) | 1 (−3; 5) | 3 (−1; 7) | 1 (−2; 5) | 1 (−2; 5) | 2 (−1; 4) | 2 (−1; 4) |
| | full model | −3 (−7; 1) | −2 (−8; 4) | −6 (−12; 0) | −5 (−13; 3) | −5 (−13; 3) | −3 (−11; 5) | −1 (−6; 4) | 0 (−5; 5) | 0 (−5; 5) | 0 (−5; 6) |
| FVC (mL) | **Candle burning** | | | | | | | | | | |
| | crude model | 8 (−2; 18) | 7 (−3; 17) | 9 (−1; 19) | 8 (−2; 18) | 9 (−1; 19) | 8 (−2; 18) | 2 (0; 4) | 0 (−2; 2) | 0 (0; 0) | 0 (0; 0) |
| | model 1 | 8 (−10; 26) | 7 (−11; 25) | 10 (−8; 28) | 9 (−9; 27) | 11 (−7; 29) | 10 (−8; 28) | 0 (−3; 4) | −1 (−4; 3) | 0 (0; 1) | 0 (−1; 0) |
| | model 2 | 1 (−17; 19) | 0 (−18; 18) | 2 (−18; 22) | 1 (−19; 21) | 5 (−13; 23) | 3 (−17; 23) | 1 (−3; 4) | −1 (−4; 3) | 0 (−1; 1) | 0 (−1; 1) |
| | full model | −13 (−38; 12) | −12 (−39; 15) | −12 (−39; 15) | −11 (−38; 16) | −13 (−44; 18) | −12 (−43; 19) | **−9 (−18; −1)** | **−10 (−18; −1)** | −1 (−3; 0) | −1 (−3; 0) |

**Table S1.** *Cont.*

| **Lung Function Measure** | **Exposure Scenario** | **PMC** | | | | | | **PSC** | | **PNC** | |
|---|---|---|---|---|---|---|---|---|---|---|---|
| | | **$PM_{10}$** | | **$PM_{2.5}$** | | **$PM_1$** | | | | **<100 nm** | |
| | | **Mean (95%-CI)** | | **Mean (95%-CI)** | | **Mean (95%-CI)** | | **Mean (95%-CI)** | | **Mean (95%-CI)** | |
| | | **Post 4 h** | **Post 24 h** | **Post 4 h** | **Post 24 h** | **Post 4 h** | **Post 24 h** | **Post 4 h** | **Post 24 h** | **Post 4 h** | **Post 24 h** |
| **FVC (mL)** | **Toasting bread** | | | | | | | | | | |
| | crude model | 0 (−2; 2) | −1 (−3; 1) | 4 (−2; 10) | 1 (−5; 7) | **14 (4; 24)** | **14 (4; 24)** | **3 (1; 5)** | 2 (0; 5) | 1 (0; 1) | 0 (0; 1) |
| | model 1 | 0 (−2; 2) | −1 (−3; −1) | 1 (−5; 7) | −1 (−7; 5) | **18 (4; 32)** | **17 (3; 31)** | **6 (3; 9)** | **6 (2; 9)** | 2 (1; 3) | 2 (1; 3) |
| | model 2 | 0 (−2; 2) | −1 (−3; −1) | 2 (−6; 10) | −1 (−9; 7) | 15 (−1; 31) | 14 (−2; 30) | **6 (2; 9)** | **5 (1; 8)** | 2 (1; 3) | 2 (1; 3) |
| | full model | 0 (−2; 2) | −2 (−10; 6) | 1 (−7; 9) | −2 (−16; 12) | 0 (−22; 22) | 0 (−21; 19) | 1 (−7; 9) | 1 (−7; 8) | 1 (−2; 4) | 1 (−2; 4) |
| | **Frying sausages** | | | | | | | | | | |
| | crude model | 0 (−2; 2) | 0 (−2; 2) | 0 (−4; 4) | 0 (−4; 4) | 1 (−3; 5) | 0 (−4; 4) | 0 (−2; 3) | −1 (−3; 1) | 0 (−1; 2) | −1 (−2; 1) |
| | model 1 | 0 (−2; 2) | 0 (−2; 2) | 1 (−3; 5) | 0 (−4; 4) | 1 (−3; 5) | 1 (−3; 5) | 0 (−3; 3) | −1 (−4; 2) | 0 (−3; 2) | −1 (−3; 1) |
| | model 2 | 0 (−2; 2) | 0 (−2; 2) | 0 (−4; 4) | 0 (−4; 4) | 1 (−3; 5) | 1 (−3; 5) | 0 (−2; 3) | −1 (−4; 2) | 0 (−2; 2) | −1 (−3; 1) |
| | full model | 2 (−2; 6) | 1 (−3; 5) | 3 (−3; 9) | 2 (−4; 8) | 5 (−1; 11) | 4 (−4; 12) | 3 (−1; 7) | 2 (−3; 7) | 4 (−1; 8) | 3 (−2; 8) |
| **$FEV_1$/FVC (%); Percentage Point * 1000** | **Candles burning** | | | | | | | | | | |
| | crude model | 0 (−2; 2) | 0 (−2; 2) | 0 (−2; 2) | 0 (−2; 2) | 0 (−2; 2) | 0 (−2; 2) | 0 (0; 0) | 0 (0; 0) | 0 (0; 0) | 0 (0; 0) |
| | model 1 | −2 (−4; 0) | −2 (−4; 0) | −2 (−4; 0) | −2 (−4; 0) | −2 (−4; 0) | −2 (−4; 0) | 0 (−1; 0) | 0 (−1; 0) | 0 (0; 0) | 0 (0; 0) |
| | model 2 | −2 (−4; 0) | −2 (−4; 0) | −2 (−4; 0) | −2 (−4; 0) | −2 (−4; 0) | −2 (−4; 0) | 0 (−1; 0) | 0 (−1; 0) | 0 (0; 0) | 0 (0; 0) |
| | full model | −2 (−6; 2) | −3 (−7; 1) | −3 (−7; 1) | −3 (−7; 1) | −3 (−7; 1) | −4 (−8; 0) | −1 (−2; 0) | 1 (−2; 0) | 0 (0; 0) | 0 (0; 0) |
| | **Toasting bread** | | | | | | | | | | |
| | crude model | 0 (0; 0) | 0 (0; 0) | 0 (0; 0) | 0 (0; 0) | 0 (−2; 2) | 0 (−2; 2) | 0 (0; 0) | 0 (0; 0) | 0 (0; 0) | 0 (0; 0) |
| | model 1 | 0 (0; 0) | 0 (0; 0) | 0 (0; 0) | 0 (0; 0) | −2 (−4; 0) | 2 (−4; 0) | 0 (−1; 0) | 0 (−1; 0) | 0 (0; 0) | 0 (0; 0) |
| | model 2 | 0 (0; 0) | 0 (0; 0) | 0 (0; 0) | 0 (0; 0) | 2 (−4; 0) | 2 (−4; 0) | 0 (−1; 0) | 0 (−1; 0) | 0 (0; 0) | 0 (0; 0) |
| | full model | 0 (0; 0) | 0 (0; 0) | 0 (0; 0) | 0 (−2; 2) | −1 (−3; 1) | −1 (−3; 1) | −1 (−2; 0) | −1 (−2; 0) | 0 (0; 0) | 0 (0; 0) |

**Table S1.** *Cont.*

| Lung Function Measure | Exposure Scenario | PMC | | | | | | PSC | | PNC | |
|---|---|---|---|---|---|---|---|---|---|---|---|
| | | $PM_{10}$ | | $PM_{2.5}$ | | $PM_1$ | | | | <100 nm | |
| | | Mean (95%-CI) | | Mean (95%-CI) | | Mean (95%-CI) | | Mean (95%-CI) | | Mean (95%-CI) | |
| | | Post 4 h | Post 24 h | Post 4 h | Post 24 h | Post 4 h | Post 24 h | Post 4 h | Post 24 h | Post 4 h | Post 24 h |
| **$FEV_1$/FVC (%); Percentage Point * 1000** | **Frying sausages** | | | | | | | | | | |
| | crude model | 0 (0; 0) | 0 (0; 0) | 0 (0; 0) | 0 (0; 0) | 0 (0; 0) | 0 (0; 0) | 0 (0; 0) | 0 (0; 1) | 0 (0; 0) | 0 (0; 0) |
| | model 1 | 0 (0; 0) | 0 (0; 0) | 0 (0; 0) | 0 (0; 0) | 0 (0; 0) | 0 (0; 0) | 0 (0; 1) | 0 (0; 1) | 0 (0; 1) | 0 (0; 1) |
| | model 2 | 0 (0; 0) | 0 (0; 0) | 0 (0; 0) | 0 (0; 0) | 0 (0; 0) | 0 (0;0) | 0 (0; 1) | 0 (0; 1) | 0 (0; 1) | 0 (0; 1) |
| | full model | −1 (−1; −1) | −1 (−1; −1) | −2 (−4; 0) | −2 (−4; 0) | −2 (−4; 0) | −2 (−4; 0) | −1 (−2; 0) | 0 (−1; 0) | −1 (−2; 0) | −1 (−1; 0) |
| **$MEF_{25\%–75\%}$ (mL/s)** | **Candles burning** | | | | | | | | | | |
| | crude model | 9 (−7; 25) | −3 (−19; 13) | 10 (−6; 26) | −2 (−18; 14) | 10 (−6; 26) | −2 (−20; 16) | 2 (−1; 6) | 1 (−3; 4) | 0 (0; 1) | 0 (0; 1) |
| | model 1 | −9 (−40; 22) | −21 (−52; 10) | −8 (−39; 23) | −21 (−52; 10) | −7 (−38; 24) | −19 (−50; 12) | −1 (−7; 5) | −3 (−9; 4) | 0 (−1; 2) | 0 (−1; 1) |
| | model 2 | −17 (−50; 16) | −29 (−62; 4) | −17 (−50; 16) | −29 (−64; 6) | −15 (−48; 18) | −27 (−60; 6) | −1 (−8; 5) | −3 (−9; 4) | 0 (−1; 2) | 0 (−1; 2) |
| | full model | −23 (−70; 24) | −37 (−84; 10) | −26 (−75; 23) | −39 (−88; 10) | −31 (−84; 22) | −45 (−100; 10) | −13 (−28;2) | −14 (−29;1) | 1 (−2; 4) | 1 (−2; 4) |
| | **Toasting bread** | | | | | | | | | | |
| | crude model | 1 (−3; 5) | 0 (−4; 4) | 5 (−5; 15) | 0 (−10; 10) | 11 (−7; 29) | 2 (−16; 20) | 3 (−1; 7) | 1 (−3; 5) | 1 (0; 2) | 0 (0; 1) |
| | model 1 | 0 (−4; 4) | −1 (−5; 3) | −1 (−13; 11) | −6 (−18; 6) | −9 (−34; 16) | −18 (−43; 7) | 0 (−5; 6) | −2 (−7; 4) | 0 (−1; 2) | 0 (−2; 2) |
| | model 2 | 0 (−4; 4) | −1 (−5; 3) | 1 (−11; 13) | −4 (−16; 8) | −6 (−33; 21) | −12 (−39; 15) | 1 (−5; 7) | 0 (−6; 6) | 1 (−1; 3) | 1 (−1; 3) |
| | full model | 1 (−3; 5) | 0 (−14; 14) | 1 (−11; 13) | −6 (−30; 18) | −14 (−49; 21) | −22 (−57; 13) | −8 (−22;6) | −9 (−23;5) | 1 (−4; 6) | 1 (−4; 6) |
| | **Frying sausages** | | | | | | | | | | |
| | crude model | 0 (−6; 6) | 2 (−4; 8) | −1 (−9; 7) | 1 (−7; 9) | 0 (−8; 8) | 2 (−8; 12) | 0 (−5; 6) | 1 (−4; 7) | 1 (−3; 4) | 1 (−3; 4) |
| | model 1 | 2 (−6; 10) | 3 (−5; 11) | 0 (−10; 10) | 2 (−8; 12) | 1 (−9; 11) | 4 (−8; 16) | 2 (−5; 9) | 3 (−4; 10) | 3 (−2; 9) | 4 (−2; 9) |
| | model 2 | 2 (−6; 10) | 3 (−5; 11) | 0 (−10; 10) | 2 (−8; 12) | 1 (−9; 11) | 4 (−8; 16) | 2 (−5; 9) | 3 (−4; 10) | 4 (−2; 9) | 4 (−1; 10) |
| | full model | **−11 (−21; −1)** | −8 (−20; 4) | **−23 (−37; −9)** | **−20 (−36;−4)** | **−22 (−38; −6)** | −19 (−39; 1) | −8 (−18; 3) | −5 (−17; 7) | −7 (−18; 5) | −5 (−17; 6) |

Notes: model 1: adjusted for age, height and sex; model 2: adjusted for age, height, sex, temperature and humidity; full model: adjusted for age, height, sex, temperature, humidity, travel time and means of transportation; Abbreviations: FEV1: forced expiratory volume at 1 s, FVC: forced vital capacity, $MEF_{25\%–75\%}$: averaged forced expiratory flow between the full expiration of 25% and 75% of the total FVC, CI: confidence interval; marked in bold: effect estimates where CI did not include the null effect. PMC: particle mass concentration, PSC: particle surface concentration, PNC: particle number concentration, LDSA: lung deposited surface area.

© 2014 by the authors; licensee MDPI, Basel, Switzerland. This article is an open access article distributed under the terms and conditions of the Creative Commons Attribution license (http://creativecommons.org/licenses/by/3.0/).
